# Active play and screen time in US children aged 4 to 11 years in relation to sociodemographic and weight status characteristics: a nationally representative cross-sectional analysis

**DOI:** 10.1186/1471-2458-8-366

**Published:** 2008-10-22

**Authors:** Sarah E Anderson, Christina D Economos, Aviva Must

**Affiliations:** 1 Division of Epidemiology, The Ohio State University College of Public Health, Columbus, Ohio, USA; 2 Gerald J. and Dorothy R. Friedman School of Nutrition Science and Policy, Tufts University, Boston, Massachusetts, USA; 3 Department of Public Health and Family Medicine, Tufts University School of Medicine, Boston, Massachusetts, USA

## Abstract

**Background:**

The high prevalence of childhood obesity underscores the importance of monitoring population trends in children's activity and screen time, and describing associations with child age, gender, race/ethnicity, and weight status. Our objective was to estimate the proportion of young children in the US who have low levels of active play or high levels of screen time, or who have both these behaviors, and to describe associations with age, gender, race/ethnicity, and weight status.

**Methods:**

We analyzed data collected during the National Health and Nutrition Examination Surveys 2001–2004, a US nationally representative cross-sectional study. We studied 2964 children aged 4.00 to 11.99 years. Our main outcomes were reported weekly times that the child played or exercised hard enough to sweat or breathe hard (active play), daily hours the child watched television/videos, used computers, or played computer games (screen time), and the combination of low active play and high screen time. Low active play was defined as active play 6 times or less per week. High screen time was defined as more than 2 hours per day. We accounted for the complex survey design in analyses and report proportions and 95% confidence intervals. We used Wald Chi-square to test for differences between proportions. To identify factors associated with low active play and high screen time, we used multivariate logistic regression.

**Results:**

Of US children aged 4 to 11 years, 37.3% (95% confidence interval, 34.1% to 40.4%) had low levels of active play, 65.0% (95% CI, 61.4% to 68.5%) had high screen time, and 26.3% (95% CI, 23.8% to 28.9%) had both these behaviors. Characteristics associated with a higher probability of simultaneously having low active play and high screen time were older age, female gender, non-Hispanic black race/ethnicity, and having a BMI-for-age ≥95^th ^percentile of the CDC growth reference.

**Conclusion:**

Many young children in the US are reported to have physical activity and screen time behaviors that are inconsistent with recommendations for healthy pediatric development. Children who are overweight, approaching adolescence, girls, and non-Hispanic blacks may benefit most from public health policies and programs aimed at these behaviors.

## Background

The prevention of childhood obesity is a public health priority in the United States [1]. Recommendations for the prevention of childhood obesity call for children to be active daily and to spend less time in sedentary pursuits, such as watching television and videos and playing video and computer games, collectively known as screen time [2-6]. The American Academy of Pediatrics recommend that children accumulate no more than 2 hours per day of screen time [3,5], and the Centers for Disease Control and Prevention recommend that children be active daily for at least 60 minutes [7].

Few nationally representative analyses have been published describing relationships between sociodemographic characteristics and physical activity in children under age 12 [8-11], and we are unaware of published US estimates of levels of activity in children younger than 6 years old. A recent analysis of accelerometry data from the US National Health and Nutrition Examination Survey (NHANES) 2003–2004 found differences by gender and race/ethnicity in activity level among 6 to 15 year old children [11]. Analyses from NHANES III (1988–1994) of 8–16 year old children's reported bouts of vigorous physical activity per week also found differences in levels of physical activity relative to child age, gender, and race/ethnicity [8]. Adolescent boys were more likely than girls to engage in vigorous activity, but at younger ages no difference by gender was observed; non-Hispanic white children reported more bouts of vigorous activity per week than non-Hispanic black or Mexican American children [8]. Among 9–13 year old US youth surveyed by telephone in 2002 [9], participation in organized physical activity was related to race/ethnicity, parental education, and parental income with Hispanic and non-Hispanic black youth less likely than non-Hispanic white youth to have participated in organized physical activity in the preceding 7 days, and greater parental education and income being associated with greater likelihood of the child having participated in organized physical activity [10]. Participation in *organized *physical activity was not related to gender or age, but boys were significantly more likely than girls to report having participated in free-time physical activity during the prior week (74.1% vs 80.5%) [10].

Associations between television viewing, media use, and children's sociodemographic and weight status characteristics have been studied more frequently [12-17]. Many studies have found that television viewing time and computer use is similar for boys and girls; however boys are more likely than girls to play video games, and boys may be somewhat more likely than girls to have very high screen times (>4 hours per day) [15].

Sedentary behavior is not simply the inverse of physical activity [18-20], and children who are highly physically active may also spend a lot of time in sedentary activities. Thus it is important to understand the prevalence and sociodemographic correlates of low levels of physical activity and high levels of sedentary behaviour individually, as well as in combination. Very little is known about how risk behaviors, such as low levels of physical activity or high levels of sedentary behavior, cluster together [21]. We are unaware of published reports representative of US young children that describe the proportion of children who have the combination of low levels of activity and high levels of sedentary behaviour.

The high prevalence of childhood obesity underscores the importance of monitoring population trends in children's activity and screen time, and describing associations with child age, gender, race/ethnicity, and weight status. Young children's physical, social, emotional, and cognitive development is enhanced by active play, particularly unstructured play [22]. Adequate daily activity and limited levels of sedentary behavior benefit children's cardiovascular health, coordination, fitness, and psychosocial development [23-26] in addition to lowering risk for obesity.

Our objective was to estimate the proportion of US children aged 4 to 11 years who are participating in low levels of active play, having greater than 2 hours per day of screen time, or both participating in low levels of active play *and *having more than 2 hours per day of screen time. We also sought to understand the relationship of these behaviors with child age, gender, race/ethnicity, and weight status.

## Methods

We analyzed data from the National Health and Nutrition Examination Survey (NHANES) 2001–2004. These data are representative of the civilian, non-institutionalized, US population. Data from cycles 2001–2002 and 2003–2004 were combined as recommended by NHANES analytic guidelines [27]. Survey design and operations are described in detail, and data are publicly available at . The Ohio State University biomedical IRB has determined that because data from NHANES are publicly available and participants cannot be identified, analyses of NHANES do not require IRB review.

Analyses included 2964 children aged 4.00 to 11.99 years. Children were examined in the NHANES Mobile Examination Center. A proxy reporter (typically a parent, most often the mother) responded to interview questions about the subject child; for simplicity we will say 'parent' throughout this report to indicate the proxy reporter.

### Active play

A parent reported the number of times per week their child played or exercised actively. Frequency and not duration of activity was assessed for children under age 12. The wording of the question asked was, 'Now I'd like to ask you some questions about {Child's} activities. How many times per week does {Child} play or exercise enough to make {him/her} sweat and breathe hard?'[28,29] We categorized a child as having a low level of active play if the response to this question was less than 7 times per week. The American Academy of Pediatrics (AAP) [4], the American Heart Association [6], Centers for Disease Control and Prevention (CDC) [7], and the US Department of Health and Human Services and US Department of Agriculture [30] recommend that children be physically active daily for at least sixty minutes.

### Screen time

A parent reported the average hours per day their child sat and watched television or videos, and also the time the child used a computer or played computer games. The specific questions asked were, 'Over the past 30 days, on average about how many hours per day did {Child} sit and watch TV or videos? and 'Over the past 30 days, on average about how many hours per day did {Child} use a computer or play computer games?" In each case, response options were: "less than 1 hour, 1 hour, 2 hours, 3 hours, 4 hours, 5 or more hours, or none" [28,29]. We coded a response of 'less than 1 hour' as 0.5 hours and a response of 5 or more hours as 5 hours. We summed average hours of television/videos and computers/computer games to estimate "screen time." We defined high screen time as greater than 2 hours per day.

### Weight status – BMI-for-age ≥95th

Children's weight and standing height were measured using a standardized protocol as part of the physical examination in the Mobile Examination Center. Four children, who were weighed while wearing a medical device, were excluded from analyses. Body mass index (BMI) was calculated as weight in kilograms divided by the square of height in meters. Age and gender-specific BMI percentiles were calculated using the Centers for Disease Control and Prevention's BMI-for-age growth reference [31]. We defined obesity as having a BMI z-score ≥95^th ^percentile relative to the CDC reference [5].

### Race/ethnicity

Race/ethnicity of the child was reported by the parent and classified by NHANES as Mexican American, non-Hispanic black, non-Hispanic white, other Hispanic, or other. The category 'other' includes individuals who indicated a single race/ethnicity other than those above, identified with 2 or more race/ethnicity categories (multiracial), or were missing information on race/ethnicity. In our logistic regression analyses we combined the categories 'other Hispanic' and 'other.'

### Covariates

The child's country of birth was reported by the parent; children were categorized as born in the US, or born outside the US. We used the poverty income ratio (PIR) as a measure of socioeconomic status. PIR is a measure of wealth that is based on family income in relation to US Census Bureau income thresholds for poverty; these income thresholds take into account family size and composition. Higher PIR values denote greater wealth. To protect confidentiality in NHANES, PIR is top-coded at 5 for all participants with a PIR above 5 [32].

### Analytic approach

Children were grouped by age at last birthday into age categories as follows: 4–5 year olds, 6–8 year olds, 9–11 year olds. To account for the complex survey design, sample weights were applied in all analyses. Analyses were conducted with SAS version 9.1 (SAS Institute, Cary, NC); prevalence estimates and 95% confidence intervals were calculated using PROC SURVEYMEANS and PROC SURVEYFREQ. We used Wald Chi-square to test for differences between proportions and logistic regression (PROC SURVEYLOGISTIC) to test for age-related trends. An alpha level of 0.05 was required for statistical significance. To identify sociodemographic and weight status factors associated with low levels of active play and high screen time, we used logistic regression with age (as a continuous variable), gender, race/ethnicity, weight status, poverty income ratio, and whether the child was born outside the US as predictors. We hypothesized the possibility of a number of interactions between these risk factors, and therefore we systematically tested for interactions between age and gender, gender and race/ethnicity, gender and weight status, and race/ethnicity and weight status in logistic regression models.

## Results

Demographic characteristics of US children aged 4 to 11 years are presented in Table 1. The prevalence of obesity (BMI z-score ≥95^th ^percentile) among 4–5 year old and 6–8 year old children was approximately 15%, and was almost 20% among 9–11 year old children.

**Table 1:** Characteristics of US children age 4 to 11 years in NHANES 2001–2004*

	**Age category ^‡^**			
	**4 to 11 years overall**	**4–5 years**	**6–8 years**	**9–11 years**
**n ^§^**	2964	777	1109	1078
**Mean age (months)**	96.1	59.6	89.7	125.5
**% male**	50.6%	48.2%	50.4%	52.3%
**% Non-Hispanic white**	57.8%	57.3%	57.2%	58.5%
**% Non-Hispanic black**	15.5%	14.4%	15.5%	16.3%
**% Mexican-American**	13.4%	14.3%	13.4%	12.9%
**Median Poverty income ratio**	2.0	1.9	2.0	2.1
**% Born in US**	95.0%	95.7%	94.9%	95.8%
**% Obese^†^**	16.9%	15.2%	15.1%	19.6%
**Mean BMI**	18.0	16.4	17.3	19.8

Abbreviations: BMI, body mass index (calculated as weight in kilograms divided by height in meters squared). NHANES, National Health and Nutrition Examination Survey.

*Estimates are weighted to be representative of US children age 4 to 11 years.

‡ Age at last birthday

^§^ n = unweighted sample size

† BMI ≥95^th ^percentile of the age- and gender-specific CDC BMI growth charts

The number of times per week that children in the sample were reported to play or exercise hard enough to sweat or breathe hard (active play) ranged from a minimum of 0 to a maximum of 77. Weighted to be representative of children aged 4 to 11 years in the US, ten percent of children engaged in this level of active play two or fewer times per week, and ten percent of children engaged in this level of active play ten or more times per week. Table 2 presents the median (25^th ^percentile, 75^th ^percentile) and mean (95% confidence interval) of the times per week of active play by gender and age category. For all age-gender combinations the median and 75^th ^percentiles were 7 times per week, except for girls aged 9–11 years where the median was 6. The median number of hours of television/videos watched per day by US children age 4 to 11 years was two hours; ten percent of children watched 30 minutes or less per day and ten percent of children watched 4 or more hours per day. Half of US children age 4 to 11 years used computers or computer games for 30 minutes or less per day, ten percent of children used computers or computer games 2 hours per day or more, and the top five percent used computers or computer games 3 or more hours per day. Table 2 presents the mean (95% confidence interval) of reported television viewing and computer/computer game use by gender and age category. The mean hours of reported computer/computer game use ranged from 0.6 hours per day for 4–5 year old girls to 1.2 hours per day for 9–11 year old boys. The correlation between active play and screen time was -0.08 (Spearman r, p = 0.001) for boys and -0.02 (Spearman r, p = 0.34) for girls.

**Table 2:** Active play, television, and computer time by gender and age category*

	**Age category ^‡^**			
	**4 to 11 years overall**	**4–5 years**	**6–8 years**	**9–11 years**
	**Boys**			
**n ^§^**	1442	375	528	539
**Active Play (times per week) †**	7 (5, 7)	7 (5, 7)	7 (5, 7)	7 (5, 7)
Active Play (times per week)**	7.0 (6.5 – 7.4)	6.9 (6.3 – 7.4)	6.9 (6.5 – 7.3)	7.1 (6.0 – 8.1)
Television (hours per day)**	2.3 (2.2 – 2.5)	2.3 (2.0 – 2.5)	2.2 (2.0 – 2.4)	2.4 (2.2–2.6)
Computer/video games (hours per day)**	0.9 (0.9 – 1.0)	0.7 (0.6 – 0.8)	0.8 (0.8 – 0.9)	1.2 (1.0 – 1.4)

	**Girls**			
**n ^§^**	1522	402	581	539
**Active Play (times per week) †**	7 (4, 7)	7 (5, 7)	7 (4, 7)	6 (3, 7)
Active Play (times per week)**	6.4 (5.8 – 7.0)	7.3 (6.2 – 8.3)	6.2 (5.7 – 6.7)	6.2 (5.4 – 6.9)
Television (hours per day)**	2.2 (2.1 – 2.3)	2.2 (2.0 – 2.3)	2.1 (1.9 – 2.3)	2.4 (2.2 – 2.5)
Computer/video games (hours per day)**	0.7 (0.7 – 0.8)	0.6 (0.5 – 0.7)	0.7 (0.6 – 0.8)	0.8 (0.7 – 0.9)

*Parent (proxy) report of the number of times per week children were reported to play or exercise hard enough to sweat or breathe hard (active play), daily hours of television watching, and computer/video for boys and girls by age category in the National Health and Nutrition Examination Survey 2001–2004. Estimates are weighted to be representative of US children age 4 to 11 years.

‡ Age at last birthday

^§^ n = unweighted sample size

† Median (25^th ^percentile, 75^th ^percentile)

** Mean (95% Confidence Interval) calculated accounting for complex survey design

### Comparisons by gender and age

Overall, 37.3% (95% confidence interval, 34.1% to 40.4%) of US children aged 4 to 11 years were reported to play or exercise hard enough to sweat or breathe hard less than 7 times per week (low levels of active play). The proportion of boys with low levels of active play (31.4%) was lower than the proportion of girls with low levels of active play (43.4%) (Table 3). In each age category, girls were more likely than boys to have low levels of active play, although in the youngest age group (4–5 years) this difference did not reach statistical significance at an alpha level of 0.05 (p = 0.061). For boys and girls, the percentage of children with low levels of active play was higher at older ages. Sixty-five percent (95% CI, 61.4% to 68.5%) of US children 4 to 11 years old overall (67.7% of boys and 62.0% of girls) were reported to have more than 2 hours per day of screen time (high screen time). The prevalence was higher at older ages for boys and girls (Table 3). Although fewer girls than boys were reported to have >2 hours per day of screen time, these gender differences were not statistically significant within age categories. The percentage of US children 4 to 11 years old who were reported to have *both *low levels of active play and high screen time was 26.3% (95% CI, 23.8% to 28.9%) overall and was 23.7% for boys and 29.1% for girls. Girls were more likely than boys overall to have low levels of active play and high screen time, but when stratified by age category this gender difference was only statistically significant in the 6–8 year age category (18.8% boys, 26.9% girls, p = 0.009). The likelihood of this combination was greater at older ages for both boys and girls; 31.2% of boys and 37.7% of girls in the 9–11 year age category had both low levels of active play and high screen time (Table 3).

**Table 3:** Proportion of boys and girls with low active play, high screen time, or both behaviors*

**Gender**		
	**Boys**	**Girls**
Percentage (95% confidence interval) ^†^		
**Low active play ^§^**		
*4 to 11 years overall*	31.4% (28.0% – 34.7%)	43.4% (39.3% – 47.6%)***
4–5 years	27.2% (21.2% – 33.2%)	35.1% (29.5% – 40.6%)^+^
6–8 years	25.7% (21.5% – 30.0%)	39.4% (34.3% – 44.6%)***
9–11 years	39.3% (32.0% – 46.6%)	52.9% (45.6% – 60.2%)***
**p trend age ^§§^**	0.008	<0.0001
**High screen time ‡**		
*4 to 11 years overall*	67.7% (63.6% – 72.1%)	62.0% (58.1% – 65.8%)***
4–5 years	60.6% (53.0% – 68.2%)	55.6% (48.4% – 62.9%)
6–8 years	65.4% (60.0% – 70.8%)	62.0% (55.9% – 68.2%)
9–11 years	74.4% (68.3% – 80.6%)	66.3% (61.0% – 71.6%)^+^
**p trend age ^§§^**	<0.0001	0.011
**Both low active play & high screen time**		
*4 to 11 years overall*	23.7% (21.0% – 26.3%)	29.1% (25.5% – 32.7%)***
4–5 years	18.9% (13.2% – 24.6%)	19.4% (14.7% – 24.2%)
6–8 years	18.8% (15.4% – 22.2%)	26.9% (22.0% – 31.8%)***
9–11 years	31.2% (24.9% – 37.5%)	37.7% (31.0% – 44.3%)
**p trend age ^§§^**	0.004	<0.0001

*Proportion of boys and girls with low levels of active play, and/or greater than 2 hours per day of screen time by age category in the National Health and Nutrition Examination Survey 2001–2004. Estimates are weighted to be representative of US children age 4 to 11 years.

† Percentage (95% Confidence Interval) calculated accounting for complex survey design.

^§ ^Low active play defined as reported to play or exercise hard enough to sweat or breathe hard less than 7 times per week.

‡ High screen time defined as >2 hours per day; Screen time includes time spent watching television or videos, and using computers or computer-games.

^§§ ^P value from logistic regressions by gender with age in years (at last birthday) as predictor, accounting for complex survey design.

+ Prevalence in girls different than prevalence in same age boys: Wald Chi-square, p < 0.1.

*** Prevalence in girls significantly different than prevalence in same age boys: Wald Chi-square, p < 0.01.

### Comparisons by weight status

Among US children 4 to 11 years old, 16.9% (95% CI, 14.8% to 18.9%) had a BMI ≥95^th ^percentile. Overall, children with a BMI ≥95^th ^percentile (obese) were more likely than children with a BMI below the 95^th ^percentile (non-obese) to have low levels of active play (42.5% vs. 36.4%, p = 0.053; borderline statistical significance), were more likely to have screen time >2 hours per day (70.9% vs. 64.3%, p = 0.040), and were more likely to have both behaviors simultaneously (32.8% vs. 25.2%, p = 0.031). Table 4 presents the percentage of children with low levels of active play, high screen time, and both behaviors stratified by gender and weight status. When stratified by gender, differences between obese and non-obese children (age 4 to 11 years overall) were statistically significant in girls for high screen time, and the combination of low active play and high screen time. Obese girls were not statistically significantly less likely than non-obese girls to have low levels of active play (47.9% vs 42.7%, p = 0.299). In boys, obese boys 4 to 11 years old were more likely than non-obese boys to have low levels of active play (38.1% vs 30.1%) but this comparison failed to reach statistical significance (p = 0.099), and appeared to be driven by the 9–11 year age category in which 53.3% (95% CI, 40.1% to 66.4%) of obese boys had low levels of active play compared to 36.5% (95% CI, 28.6% to 44.5%) of non-obese boys. Confidence intervals for estimates in the obese subgroup are wide, particularly when stratified by age category, due to small sample sizes within these cells.

**Table 4:** Proportion with low active play, high screen time, or both behaviors by weight status*

**Weight status**		
	**BMI < 95^th ^percentile (non obese)**	**BMI ≥95^th ^percentile (obese)**
Percentage (95% confidence interval)^†^		
**Low active play ^§^**	**Boys**	
*4 to 11 years overall*	**30.1% (26.2% – 33.9%)**	**38.1% (29.2% – 47.0%)^+^**
4–5 years	25.3% (19.3% – 31.2%)	37.6% (19.9% – 55.3%)
6–8 years	26.3% (21.2% – 31.5%)	21.7% (11.4% – 32.0%)
9–11 years	36.5% (28.6% – 44.5%)	53.3% (40.1% – 66.4%)**
		
**High screen time ^‡^**		
*4 to 11 years overall*	**67.9% (62.6% – 73.3%)**	**70.0% (63.4% – 76.6%)**
4–5 years	62.8% (55.2% – 70.3%)	55.5% (40.2% – 70.6%)
6–8 years	64.2% (57.3% – 71.0%)	74.5% (64.1% – 84.9%)
9–11 years	74.6% (67.7% – 81.6%)	73.7% (63.6% – 83.8%)
		
**Both low active play & high screen time**		
*4 to 11 years overall*	**23.0% (19.3% – 26.7%)**	**28.5% (20.3% – 36.8%)**
4–5 years	17.0% (11.3% – 22.7%)	30.3% (14.6% – 46.1%)
6–8 years	19.4% (15.2% – 23.6%)	16.2% (6.9% – 25.5%)
9–11 years	29.9% (21.9% – 37.9%)	38.6% (25.9% – 51.4%)

**Low active play ^§^**	**Girls**	
*4 to 11 years overall*	**42.7% (38.0% – 47.4%)**	**47.9% (38.9% – 56.9%)**
4–5 years	36.0% (30.1% – 41.9%)	32.2% (18.3% – 46.1%)
6–8 years	38.4% (33.1% – 43.6%)	49.0% (31.2% – 66.8%)
9–11 years	52.2% (44.7% – 59.7%)	53.6% (39.8% – 67.4%)
		
**High screen time ^‡^**		
*4 to 11 years overall*	**60.7% (56.8% – 64.6%)**	72.0% (64.5% – 79.5%)***
4–5 years	55.0% (47.2% – 62.7%)	62.8% (47.7% – 77.9%)
6–8 years	60.5% (54.4% – 66.7%)	77.8% (68.4% – 87.1%)**
9–11 years	65.2% (59.1% – 71.2%)	72.0% (63.5% – 80.6%)
		
**Both low active play & high screen time**		
*4 to 11 years overall*	**27.5% (23.7% – 31.3%)**	38.1% (30.3% – 45.9%)**
4–5 years	19.3% (14.7% – 23.8%)	21.2% (6.8% – 35.6%)
6–8 years	25.1% (20.5% – 29.7%)	40.1% (23.2% – 57.0%)
9–11 years	36.0% (28.9% – 43.1%)	43.7% (31.5% – 55.9%)

Abbreviations: BMI, body mass index (calculated as weight in kilograms divided by height in meters squared).

*Proportion of boys and girls with low levels of active play, and/or greater than 2 hours per day of screen time by weight status and age category in the NHANES 2001–2004. Estimates are weighted to be representative of US children age 4 to 11 years.

† Percentage (95% Confidence Interval) calculated accounting for complex survey design

^§^ low active play defined as reported to play or exercise hard enough to sweat or breathe hard <7 times per week.

‡high screen time defined as >2 hours per day. Screen time includes time spent watching television or videos, and using computers or computer-games.

+ Prevalence in obese children different than in non-obese children of the same age and gender: Wald Chi-square, p < 0.1.

** Prevalence in obese children significantly different than in non-obese children of the same age and gender: Wald Chi-square, p < 0.05.

*** Prevalence in obese children significantly different than in non-obese children of the same age and gender: Wald Chi-square, p < 0.01.

### Comparisons by race/ethnicity

Percentages for these behaviors are tabulated in Table 5 for Mexican American, non-Hispanic white, and non-Hispanic black boys and girls by age category. Among 4 to 11 year olds overall, race/ethnicity was not statistically significantly related to the percentage of boys or girls who had low levels of active play; there was a trend among 6–8 year olds for Mexican American boys to be more likely than non-Hispanic white boys to have low levels of active play. There were statistically significant differences by race/ethnicity for high screen time in boys and girls, with a greater percentage of non-Hispanic black children than Mexican American or non-Hispanic white children reported to have >2 hours per day of screen time; 78.7% of non-Hispanic black boys and 76.2% of non-Hispanic black girls had high screen time, compared to 66.3% and 60.3% of non-Hispanic white boys and girls, respectively, and 60.6% and 56.9% of Mexican American boys and girls. There was a trend for Mexican American 6–8 year old boys to be more likely than non-Hispanic white boys (25.2% vs. 16.6%) to have both low levels of active play and high screen time, and among 4 to 11 year old girls for non-Hispanic black girls to be more likely than Mexican American girls (31.9% vs 24.7%) to have this combination of behaviors (Table 5).

**Table 5:** Proportion with low active play, high screen time, or both behaviors by race/ethnicity*

	**Mexican American**	**non-Hispanic white**	**non-Hispanic black**
		Percentage (95% confidence interval)^†^	
**Low active play ^ §^**		**Boys**	
*4 to 11 years overall*	**36.4% (30.5% – 42.2%)**	**29.8% (24.9% – 34.7%)**	**31.5% (25.9% – 37.1%)**
**4–5 years**	36.2% (23.6% – 48.7%)	23.8% (15.3% – 32.4%)	31.0% (18.8% – 43.3%)
**6–8 years**	37.1% (28.9% – 45.3%)	24.1% (18.5% – 29.6%)	28.2% (22.0% – 34.4%)^+b^
**9–11 years**	35.7% (25.8% – 45.6%)	38.8% (27.7% – 49.8%)	34.5% (27.4% – 41.6%)
			
**High screen time ^‡^**			
*4 to 11 years overall*	**60.6% (54.5% – 66.7%)**	**66.3% (61.1% – 71.4%)**	78.7% (74.6% – 82.8%)***^ac^
**4–5 years**	50.1% (40.0% – 60.2%)	62.6% (52.8% – 72.4%)	70.0% (61.4% – 77.9%)**^c^
**6–8 years**	63.4% (54.9% – 71.9%)	61.5% (54.2% – 68.9%)	76.4% (69.3% – 83.4%)**^ac^
**9–11 years**	64.4% (55.9% – 73.0%)	73.0% (64.4% – 81.6%)	85.3% (79.1% – 91.5%)***^ac^
			
**Both low active play & high screen time**			
*4 to 11 years overall*	**24.5% (20.2% – 28.9%)**	**21.8% (17.7% – 25.9%)**	**27.2% (22.2% – 32.2%)**
**4–5 years**	22.5% (12.7% – 32.3%)	15.3% (6.7% – 23.9%)	26.5% (15.9% – 37.2%)
**6–8 years**	25.2% (18.2% – 32.3%)	16.6% (11.7% – 21.6%)	23.8% (17.2% – 30.3%)^+b^
**9–11 years**	25.1% (17.8% – 32.4%)	30.6% (20.1% – 41.0%)	30.4% (24.1% – 36.7%)

**Low active play ^§^**		**Girls**	
*4 to 11 years overall*	**44.0% (37.5% – 50.4%)**	**42.5% (36.4% – 48.5%)**	**40.6% (34.8% – 46.4%)**
**4–5 years**	35.9% (26.6% – 45.2%)	34.7% (24.7% – 44.6%)	31.7% (22.4% – 41.0%)
**6–8 years**	43.6% (29.8% – 44.4%)	37.1% (29.8% – 44.4%)	37.3% (29.7% – 44.9%)
**9–11 years**	49.9% (39.0% – 60.9%)	52.5% (41.7% – 63.4%)	49.6% (41.9% – 57.3%)
			
**High screen time ^‡^**			
*4 to 11 years overall*	**56.9% (51.3% – 62.5%)**	**60.3% (55.1% – 65.5%)**	76.2% (71.6% – 80.7%)***^ac^
**4–5 years**	48.9% (40.5% – 57.3%)	53.7% (43.1% – 64.3%)	71.1% (63.2% – 79.0%)***^ac^
**6–8 years**	60.0% (52.2% – 67.3%)	59.3% (50.6% – 67.9%)	79.3% (73.9% – 84.8%)***^ac^
**9–11 years**	59.8% (51.3% – 68.2%)	65.8% (58.4% – 73.2%)	75.6% (66.1% – 85.1%)^+c^
			
**Both low active play & high screen time**			
*4 to 11 years overall*	**24.7% (20.6% – 28.8%)**	**27.8% (22.7% – 33.0%)**	**31.9% (27.9% – 36.0%)^+c^**
**4–5 years**	18.1% (11.2% – 24.9%)	18.1% (10.6% – 25.7%)	24.0% (17.1% – 30.9%)
**6–8 years**	26.6% (20.9% – 32.3%)	24.3% (17.4% – 31.1%)	30.9% (23.4% – 38.3%)
**9–11 years**	27.4% (19.6% – 35.1%)	37.3% (27.3% – 47.3%)	37.8% (30.1% – 45.5%)

Abbreviations: BMI, body mass index (calculated as weight in kilograms divided by height in meters squared).

*Proportion of boys and girls with low levels of active play, and/or greater than 2 hours per day of screen time by race/ethnicity and age category in the National Health and Nutrition Examination Survey 2001–2004. Estimates are weighted to be representative of US children age 4 to 11 years.

† Percentage (95% Confidence Interval) calculated accounting for complex survey design

^§^ low active play defined as reported to play or exercise hard enough to sweat or breathe hard less than 7 times per week.

‡ high screen time defined as >2 hours per day. Screen time includes time spent watching television or videos, and using computers or computer-games.

^+ ^Difference in prevalence by race/ethnicity within gender: Wald Chi-square, p < 0.1.

** Significant difference in prevalence by race/ethnicity within gender: Wald Chi-square, p < 0.05.

*** Significant difference in prevalence by race/ethnicity within gender: Wald Chi-square, p < 0.01.

Pair-wise comparisons p < 0.05; a = non-Hispanic white different from non-Hispanic black; b = non-Hispanic white different from Mexican American; c = non-Hispanic black different from Mexican American.

### Multivariate models: sociodemographic and weight status risk factors

We used multivariate logistic regression analysis to identify characteristics of a child being reported to have low levels of active play, or to have >2 hours per day of screen time, or to have both of these behaviors together. Parameter estimates, odds ratios, and 95% confidence limits are presented in Table 6. Interactions between age and gender, gender and race/ethnicity, gender and weight status, and race/ethnicity and weight status were tested and were not statistically significant. In a multivariate adjusted model, the following characteristics were associated with increased probability of having low levels of active play: older age, female gender, Mexican American race/ethnicity, and higher poverty income ratio (greater wealth). As predictors of having high screen time, older age, male gender, non-Hispanic black race/ethnicity, and having been born in the US were associated with increased probability. The likelihood of a child having both low levels of active play *and *high screen time was increased with older age, female gender, non-Hispanic black or 'other' race/ethnicity, obesity, and having been born in the US. Based on this latter model, a girl would be 33% (95% CI: 8% to 63%) more likely than an otherwise similar boy to have both low levels of active play and >2 hours per day of screen time. A child with a BMI ≥95^th ^percentile would be 39% (95% CI: 1% to 93%) more likely than a non-obese child to have both these behaviors.

**Table 6:** Logistic regression models predicting low active play, high screen time, or both behaviors

	**Parameter estimate**	**SE**	**P value Chi-square**	**Odds ratio**	**95% Wald Confidence Limits**
**Low active play^§^**					
Age (years)	0.128	0.029	<0.0001	1.14	1.07 – 1.20
Female †	0.547	0.103	<0.0001	1.73	1.41 – 2.12
Non-Hispanic black *	0.179	0.128	0.162	1.20	0.93 – 1.54
Mexican American *	0.472	0.127	0.0002	1.60	1.25 – 2.06
Other race/ethnicity*	0.412	0.217	0.057	1.51	0.99 – 2.31
Obese **	0.218	0.132	0.099	1.24	0.96 – 1.61
Poverty to income ratio	0.136	0.042	0.001	1.15	1.06 – 1.25
Born outside US‡	-0.307	0.263	0.243	0.74	0.45 – 1.23
**High screen time^§§^**					
Age (years)	0.104	0.022	<0.0001	1.11	1.06 – 1.16
Female †	-0.265	0.098	0.007	0.77	0.63 – 0.93
Non-Hispanic black *	0.694	0.124	<0.0001	2.00	1.57 – 2.55
Mexican American *	-0.130	0.121	0.283	0.88	0.69 – 1.11
Other race/ethnicity*	0.134	0.167	0.421	1.14	0.83 – 1.59
Obese **	0.226	0.160	0.158	1.25	0.92 – 1.71
Poverty to income ratio	-0.010	0.040	0.811	0.99	0.92 – 1.07
Born outside US‡	-0.682	0.221	0.002	0.51	0.33 – 0.78
**Both low active play & high screen time**					
Age (years)	0.144	0.033	<0.0001	1.16	1.08 – 1.23
Female †	0.283	0.105	0.007	1.33	1.08 – 1.63
Non-Hispanic black +	0.358	0.137	0.009	1.43	1.09 – 1.87
Mexican American +	0.223	0.139	0.109	1.25	0.95 – 1.64
Other race/ethnicity+	0.517	0.233	0.026	1.68	1.06 – 2.65
Obese **	0.330	0.166	0.047	1.39	1.01 – 1.93
Poverty to income ratio	0.071	0.045	0.115	1.07	0.98 – 1.17
Born outside US‡	-0.592	0.236	0.012	0.55	0.35 – 0.88

Abbreviations: SE, Standard error.

*Estimates account for complex sample design and are weighted to be representative of US children age 4 to 11 years.

^§ ^Low active play defined as reported to play or exercise hard enough to sweat or breathe hard less than 7 times per week.

^§§ ^High screen time defined as >2 hours per day. Screen time includes time spent watching television or videos, and using computers or computer-games.

† Compared to male

+ Compared to non-Hispanic white

** BMI ≥95^th ^percentile for age and gender compared to BMI < 95^th ^percentile for age and gender

‡ Born outside US compared to born in the US

## Discussion

We have analyzed nationally representative data collected in the National Health and Nutrition Examination Surveys 2001–2004; these descriptive analyses provide estimates of the proportion of US children (age 4 to 11 years) by age, gender, race/ethnicity, and weight status, who have low levels of active play and high levels of screen time. As compared to adolescents, for younger children less is known about of these behaviors at the national level. In addition we provide estimates of the proportion of US children who *both *have low levels of active play and high screen time. Little is known about how frequently these behaviors cluster in young children and we are unaware of previously published nationally representative estimates describing the proportion and sociodemographic characteristics of children who have low levels of activity in combination with high levels of sedentary behavior.

Our analyses suggest that a substantial proportion of young children in the US are inadequately active *and *highly sedentary. Low levels of active play and high levels of screen time in young children are likely contributors to childhood obesity rates in the US. When these behaviors are combined, as in almost 20% of 4–5 year old boys and girls and over 35% of 9–11 year old girls, the positive energy balance required for excess weight gain is particularly likely to result [33]. That sedentary behavior and physical activity are not strongly negatively correlated is perhaps surprising, and whether they are correlated depends upon how both are measured, but having a high level of sedentary behavior does not presume that one has a low level of physical activity [19,20]. Consistent with this, in our analyses the correlation between active play and screen time was small (-0.08 in boys and -0.02 in girls). In addition, other than age and gender, the sociodemographic factors most strongly associated with low active play were not the same as those associated with high screen time. Based on our multivariate models, Mexican American children had increased likelihood of low active play whereas non-Hispanic black children had increased likelihood of high screen time. In contrast to other studies [18], we observed that higher wealth (poverty to income ratio) was associated with increased likelihood of low active play, but not associated with likelihood of high screen time.

Independent of age, gender, race/ethnicity, poverty to income ratio, and non-US country of origin, children with a BMI z-score above the 95^th ^percentile were more likely to have the combination of low levels of active play and high screen time. Although, cross-sectional analyses such as these do not provide information about cause and effect, they do indicate co-occurrence; regardless of causality, children with high BMIs are at higher risk for obesity as adults [34,35], and the combination of low levels of active play and high levels of screen time may increase the likelihood of obesity persistence into adulthood.

Our findings are consistent with a growing body of research demonstrating high levels of television viewing and media use in children in the US and other developed countries. Our study indicated that 65% of 4 to 11 year old children in the US spend, on average, more than 2 hours per day in front of a television or computer screen (high screen time). Even for preschool children (4–5 years), 60% of boys and 55% of girls had high screen time. By age 10, this proportion was above 70% in both genders. This is comparable to estimates from a study of 10 to 12 year old children in Melbourne Australia, in which 61% of boys and 57% of girls reported watching, on average, more than 2 hours per day of television [36]. Likewise, in a nationally representative survey of Canadian adolescents, 59% of girls and 66% of boys watched more than 2 hours per day of television [14]. Analyses of the US Third National Health and Nutrition Examination Survey 1988–1994, indicated that 26% of 8 to 16 year old children watched 4 or more hours of television per day [8]. Similarly, in the Health Behaviour in School-aged Children study (1988), the proportion of 11 to 15 year old youth watching more than 4 hours per day of television was approximately 19% in Austria, 21% in Norway, 23% in Finland, 28% in Hungary and Scotland, and 37% in Wales [37]. In a recent analysis of NHANES 1999–2002, Mendoza et al. examined preschooler's prior day media use and reported that 31.4% of 2 to 5 year old children had watched greater than 2 hours per day of television/videos on the previous day; when computer use was included 37.3% of preschoolers had greater than 2 hours per day of screen time [16]. Consistent with this, in a US nationally representative telephone survey conducted in 2005, 44% of 3 to 4 year olds and 30% of 5 to 6 year olds had media use greater than 2 hours per day [17]. These estimates are substantially lower than our estimate for 4 to 5 year old children of 58% having greater than 2 hours per day of screen time; this difference may be due in part to how screen time was assessed. In the studies by Mendoza et al. [16] and Vandewater et al. [17] parents were asked to report how much television, video, and computer time their child had *on the previous day*, whereas our estimates are based on questions asking parents to report the *average *hours per day their child watched or used a computer during the *previous 30 days*. Between NHANES 1999–2000 and NHANES 2001–2002 the time period referenced in the questions about children's television viewing and computer/video use changed from the prior day to the average of the previous 30 days [38].

In our analyses of 4 to 11 year old children, age was related to screen time, with older children having greater daily screen time than younger children. This is consistent with other studies of preadolescent children [15,39,40]. Research suggests that the association between age and screen time may be curvilinear with increasing levels in early childhood, a peak at about age 9 to 12 years and decreasing levels in adolescence [15]. Gender was related weakly to screen time, with 67.7% of boys and 62.0% of girls having more than 2 hours per week; but this gender difference was more pronounced in the 9–11 year olds than in the 4–5 year olds or 6–8 year olds. Estimates from our multivariate model indicate that a girl was 77% as likely to have high screen time as an otherwise similar boy. Some of this gender difference in screen time may be due to computer/video game use rather than television viewing [36]. Consistent with other studies [41], children who were born outside the US were less likely to have high screen time.

Many studies have shown an association between screen time and increased adiposity in children [8,12,13,16,20]. Evidence supports a number of different, but potentially correlated, mechanisms by which screen time may influence risk for obesity; these include exposing children to a greater degree of food advertising and thus potentially influencing their intake of energy dense, nutrient poor, snack foods [42-44], encouraging greater food consumption when children eat while viewing television [45,46], and impacting sleep quality or quantity [47,48]. Whether or not screen time displaces physical activity is controversial [19,49]. In our analyses, a statistically significantly higher proportion of obese girls had high screen time compared to non-obese girls (72% vs 61%) and this was particularly true in the 6–8 year age category in which 78% of obese girls had high screen time compared to 61% of non-obese girls. In boys, the difference between obese and non-obese boys overall (70% vs. 68%) was considerably smaller and not statistically significant. Interestingly, as in the girls, it was the 6–8 year old age category for boys in which the difference in prevalence by weight status was largest (75% of obese compared to 64% of non-obese 6–8 year old boys); however this difference in boys is not statistically significant, and thus might be due to sampling error. We speculate that the influence of obesity on a child's screen time may be different by gender because of societal differences in how obesity is perceived in boys and girls; stigmatization of obesity is greater in girls, and girls are more likely than boys to have concerns about appearance [50]. It is possible that obese girls are thus more likely to socially withdraw and choose activities, such as screen time, that are typically more solitary. However, our analyses are cross-sectional and as such provide no information about whether or not television viewing causes obesity. In our multivariate model, obesity was associated with increased likelihood of high screen time; however this was not a statistically significant association when controlling for the other variables in the model.

Estimates of physical activity from nationally representative samples of children under age 12 years are few; these estimates for US adolescents are more available because physical activity is assessed in the Youth Risk Behavior Surveillance Survey [51], and was assessed in the National Longitudinal Survey of Adolescent Health [52]. Comparison of estimates from our analyses of levels of active play to the few estimates of physical activity that are available for preadolescent children in the US is difficult because of differences in question wording, respondent (parent or child), and when the data were collected. In 2002, a nationally representative telephone survey of school-age (9 to 13 years) youth assessed self-reported participation in organized and unorganized physical activity and found that 61.5% of these children had not engaged in any organized physical activity (outside of school) in the previous 7 days, and 22.6% had not engaged in any unorganized physical activity [9,10]. Published analyses of NHANES III (1988–1994) indicate that 80% of 8 to 16 year old US children had engaged in 3 or more bouts of vigorous activity in the previous week [8]. Recently published analyses of physical activity as measured by accelerometry in US 6 to 11 year old children indicate that 49% of boys and 35% of girls in this age range accumulate 60 minutes or more of moderate or vigorous activity on at least 5 out of 7 days [11]. These findings are not inconsistent with our estimates of low levels of active play among 26% and 39% of 6–8 year old boys and girls respectively, and 39% and 53% of 9–11 year old boys and girls. International comparisons of the level of physical activity among 10 to 16 year old youth in 34 countries indicate that US youth may be more active than youth in other developed countries; 49.5% of US youth in the 2001–2002 Health Behaviour in School-aged Children Study reported engaging in at least 60 minutes of physical activity on 5 or more days per week, and this percentage was higher than any other country (Ireland was next highest at 47.2% of youth and France was lowest at 19.3%) [53].

In adolescents, age-related declines in physical activity among girls and boys have been observed repeatedly [54-58]. Younger children have been studied less frequently, but evidence suggests that age-related declines in physical activity may begin before adolescence. Trost et al [59] studied 1110 first- through twelfth-grade public school students in Western Massachusetts (US) and found that physical activity was highest at the youngest grades and decreased over the older grades. In a prospective study of 379 Iowa (US) children observed at approximately age 5.5 years and again 3 years later, physical activity as assessed by accelerometery was higher at baseline than at follow-up [39]. Troiano et al [11] analyzed physical activity as assessed by accelerometery in NHANES 2003–2004 and found that both the average accelerometery counts per minute and minutes per day in moderate or vigorous activity were higher for 6–11 year old boys than 12–15 year old boys, with a similar pattern by age for girls. In our analyses of NHANES 2001–2004, a larger sample size allowed for narrower age categories. We observed that 9–11 year old boys were more likely than 6–8 year old boys to have low levels of active play, and that the same was true for girls. In the European Youth Heart Study, 9 year old and 15 year old children from Denmark, Estonia, and Portugal were assessed, and the older children had lower levels of physical activity than the younger children irrespective of country or gender [60]. In contrast, analyses of US children studied in 1988–1994 (NHANES III) indicated that for boys and girls 11–13 year olds were somewhat *more *active than 8–10 year olds [8]. We observed that boys were more active than girls across the age range we analyzed, and this is consistent with many previous studies [11,55,57-60]. In our multivariate model older age and female gender were both associated with increased likelihood of low active play.

Assessment of physical activity in population-based studies of children presents substantial challenges [61]. Objective monitoring of physical activity, as occurs with accelerometry, is beyond the scope of most studies and not without its own methodological issues [61,62]. Beginning in 2003–2004, NHANES included assessment of activity by accelerometry for participants over age 6 years [11]. Questionnaire-based methods are more common, and rely on self- or proxy-reports (the latter most often by a parent). Evidence for the validity of parent reports of children's physical activity is quite limited. A comparison of parent and child-reports of physical activity in seventh- through twelfth-grade youth found moderate agreement in the report of physical activity correlates [63]. In a recent study comparing early elementary school-aged children's responses to those of their parents, moderate agreement was found for reporting of commonly played games and activities and transportation to school [64]. Young children are generally unable to accurately report the number of minutes spent in physical activity [65], and therefore parent reports or objective observations are necessary. In NHANES, subjects age 12 years and older are asked a series of questions regarding their frequency, duration, intensity, and type of physical activity, whereas for younger children, a parent is asked to make a much cruder assessment of frequency of bouts of moderate to vigorous activity. In contrast, the screen time items query the number of hours of television or video viewing, or using the computer. This approach makes good sense for television viewing, where TV is generally consumed in 30-minute portions [66]. However, this may not be true for other aspects of screen time, and little empirical data are available to assess the accuracy of parental reports of children's computer or video use.

Several limitations of our analyses are noteworthy. First, our estimates of the proportion of children who have low levels of active play are likely to be underestimates. We categorized children as having low levels of active play if their parent's response to the question, ''How many times per week does {Child} play or exercise enough to make {him/her} sweat and breathe hard'' was 6 or fewer. However, some children who had 7 or more times of active play per week may not play actively *daily*; for example, a child could be play actively 7 times on 1 day and not again the remainder of the week. Second, active play and screen time were not measured directly; a proxy (generally a parent) reported the frequency with which their child played or exercised hard enough to sweat or breathe hard, and the hours per day their child watched television and videos or played on a computer. Research on social desirability [67] would suggest that it is more likely that a parent would over-report activity, and under-report screen time than the reverse. For all these reasons, we believe that our estimates of the proportion of children who have low levels of active play or more than 2 hours per day of screen time are conservative. Third, some aspects of screen time may not have been captured; video games were not asked about directly, and it is not clear whether parents would include video games when responding to the question about computers and computer games. In addition, our analyses are cross-sectional, and thus causal associations cannot be inferred between the sociodemographic and weight status factors we studied in relation to low active play, high screen time, and the combination of low active play and high screen time.

## Conclusion

Many childhood obesity prevention policies call for children to be physically active daily and to limit their television viewing and other screen time. It is worrisome that many young children may not be engaging in active play every day. Perhaps more worrisome is the combination of high screen time and low levels of active play. When high screen time is combined with low levels of active play it raises questions about how young children are spending their time, and the impact this may have on their development and ultimately the health of our society. Our analyses make clear the potential for all segments of the population of 4 to 11 year old children in the US to become more active and less sedentary. Children who are approaching adolescence, girls, and non-Hispanic blacks are most likely to have low levels of active play and high screen time, and may benefit most from public health policies and programs aimed at these behaviors.

## Competing interests

The authors declare that they have no competing interests.

## Authors' contributions

SEA participated in the design and conception of the research, led the analysis and interpretation of data, and drafted the manuscript. CDE contributed to the interpretation of data and the development of the manuscript. AM participated in the conception and design of the research, interpretation of data, and drafting of the manuscript. All authors read and approved the final manuscript.

## Pre-publication history

The pre-publication history for this paper can be accessed here:


